# Antimicrobial Approaches for Textiles: From Research to Market

**DOI:** 10.3390/ma9060498

**Published:** 2016-06-21

**Authors:** Diana Santos Morais, Rui Miranda Guedes, Maria Ascensão Lopes

**Affiliations:** 1 CEMUC, Departamento de Engenharia Metalúrgica e Materiais, Faculdade de Engenharia, Universidade do Porto, Rua Dr. Roberto Frias, Porto 4200-465, Portugal; diana.s.morais@gmail.com; 2 INEGI-Instituto de Engenharia Mecânica e Gestão Industrial, Rua Dr. Roberto Frias, Porto 4200-465, Portugal; rmguedes@fe.up.pt; 3 Departamento de Engenharia Mecânica Faculdade de Engenharia, Universidade do Porto, Rua Dr. Roberto Frias, Porto 4200-465, Portugal

**Keywords:** textiles, antimicrobial, antibacterial, durability, environment impact, health impact

## Abstract

The large surface area and ability to retain moisture of textile structures enable microorganisms’ growth, which causes a range of undesirable effects, not only on the textile itself, but also on the user. Due to the public health awareness of the pathogenic effects on personal hygiene and associated health risks, over the last few years, intensive research has been promoted in order to minimize microbes’ growth on textiles. Therefore, to impart an antimicrobial ability to textiles, different approaches have been studied, being mainly divided into the inclusion of antimicrobial agents in the textile polymeric fibers or their grafting onto the polymer surface. Regarding the antimicrobial agents, different types have been used, such as quaternary ammonium compounds, triclosan, metal salts, polybiguanides or even natural polymers. Any antimicrobial treatment performed on a textile, besides being efficient against microorganisms, must be non-toxic to the consumer and to the environment. This review mainly intends to provide an overview of antimicrobial agents and treatments that can be performed to produce antimicrobial textiles, using chemical or physical approaches, which are under development or already commercially available in the form of isolated agents or textile fibers or fabrics.

## 1. Introduction

World fiber consumption has increased over several decades; from 1950 to 2008, the per capita consumption increased from 3.7 kg to 10.4 kg, and through continuous development, it recorded in 2014 a demand of 55.2 million tons (122 billion pounds) of synthetic fibers, in addition to the natural fibers, including cotton and wool, which have a demand of 25.4 million tons [1,2]. Fiber-based textile structures play an important role in several industries throughout the world, being used every day in order to meet different purposes [3,4]. Obviously, the technological advances of textiles are mainly recognized in clothing products; however, they also play important roles in other industries, such as food packaging, domestic home furnishings, automotive textiles, air filters, water purification systems, thermal and mechanical protection, sport equipment, medical devices, healthcare and hygienic applications [4,5,6].

Due to their large surface area and ability to retain moisture, textiles are known as being conducive to microorganisms’ growth, such as bacteria and fungi, which can be found almost everywhere and are able to quickly multiply, depending on the moisture, nutrients and temperature levels [7]. Some bacteria populations may double every 20–30 min under ideal conditions (36–40 °C, pH 5–9), meaning that one single bacteria cell can increase to 1,048,576 cells in just 7 h [8]. The microorganism’s growth on textiles causes a range of undesirable effects, not only on the textile itself, but also on the user. These effects include the generation of unpleasant odor, reduction in mechanical strength, stains and discoloration and an increased likelihood of user contamination [6,7]. Therefore, due to the growing public health awareness of the pathogenic effects, over the last few years, intensive research and development have been promoted in order to minimize or even eliminate microbe’s growth on textiles. This microbial contamination is a great concern, mainly for textiles used in hospitals as medical devices or for health and hygienic care, but also in sports clothing, water purification systems, animal feed and the food industry. The infections acquired in hospitals may be caused by several species, such as *Escherichia coli*, *Klebsiella pneumonia*, *Pseudomonas aeruginosa* and *Acinetobacter baumannii*.

Therefore, as consumers are becoming increasingly aware of the implications on personal hygiene and the health risks associated with some microorganisms, the demand for antimicrobial textiles has presented a big increase over the last few years [7,9]. In 2000, it was estimated that the production of antimicrobial textiles reached about 30,000 tonnes in Western Europe and 100,000 tonnes worldwide [7]. Moreover, between 2001 and 2005, in Western Europe it was reported an annual production increase of antimicrobial textiles around 15%, being one of the fastest growing sectors of the textiles industry [7]. In a recent issue of *Performance Apparel Markets*, the report “Antimicrobial fibres, fabrics and apparel: innovative weapons against infection” referred to the global market for antimicrobial agents being expected to increase by about 12% each year between 2013 and 2018 [10].

In order to impart an antimicrobial ability to textiles, different approaches have been studied, which can be mainly divided into the inclusion of antimicrobial compounds in the polymeric fibers that can leach from the polymeric matrix, the grafting of certain moieties onto the polymer surface or the physical modification of the fibers’ surface [6,11,12]. Regarding the antimicrobial compounds, different types have been used, such as quaternary ammonium compounds, triclosan, metal salts, polybiguanides or even natural polymers [7,11,12]. Any antimicrobial treatment performed on a textile needs to satisfy different requirements besides being efficient against microorganisms, but the main challenge is the concomitant requirement of non-toxicity to the consumer, namely cytotoxicity, allergy or irritation and sensitization [7,11,13]. Moreover, microorganisms in the presence of some antimicrobial agents may become resistant and the appearance of multi-drug-resistant bacteria is increasing at a worrying rate, being for the medical world one of the biggest challenges to face [11,14]. Thus, the development of new and efficient antimicrobial treatments is still an important current topic of research, mostly regarding an alternative therapeutic strategy based on plant-derived antimicrobials [9,15,16]. In order to control the generation of resistant bacteria, not only the effective prevention and control of infections is extremely important, but also monitoring the practice and application of antimicrobial agents [14,17].

This review mainly intends to provide an overview of the different antimicrobial treatments that can be performed to produce antimicrobial textiles, using chemical or physical approaches, presenting an extensive listing of treatments under development and commercially available agents and textile fibers or fabrics. Afterwards, a detailed discussion is conducted about the environmental and public health impact due to the increasing application rates of antimicrobials agents.

## 2. Textile Antimicrobial Treatments

As already mentioned, an antimicrobial treatment performed on a textile needs to satisfy different requirements besides being efficient against microorganisms, namely to be suitable for textile processing; to present durability to laundering, dry cleaning and hot pressing; to present a favorable safety and environmental profile; and it should not harm the textile quality or appearance [7,9].

Depending on the antimicrobial agent that is intended to be used, as well the fiber type, including the composition, structure and surface texture, there are different chemical and physical approaches that have been developed or that are under development to impart antimicrobial properties to the textile [6,7]. Some approaches are based on the use of specific antimicrobial agents, which in the case of synthetic fibers may be incorporated into the polymeric matrix [7,11]. Another possibility, which can be used for synthetic and natural fibers or any textile fabric, is the application, in the finishing stage, of antimicrobial agents on the material surface [6,7]. Depending on the approach used the antimicrobial textile may act by two different ways, by contact and/or diffusion. In the case of contact, the agent is placed on the fiber and does not disperse, so it will act just if the microorganism touches the textile surface. In the case of diffusion, the agent is on the fiber surface or in the polymeric matrix, and it will migrate from the textile to the external medium to attack the microorganisms [18].

A living microbe, a bacterium or fungus, presents an outermost cell wall composed of polysaccharides, which maintains the integrity of cellular components and protects the cell from the extracellular environment [7,19,20]. Beneath the cell wall is a semipermeable membrane, which encloses the intracellular organelles, enzymes, which are responsible for chemical reactions within the cell, and nucleic acids, which store all of the genetic information. The survival and growth of microorganisms depend on the cell integrity and, consequently, proper function. Therefore, an antimicrobial chemical agent or material may be classified according to the mode of action against cells’ function or integrity. If their effect happens just due to the inhibition of cell growth, they present a biostatic effect, but if they can kill the microorganisms, their effect is called biocidal [7,21].

### 2.1. Antimicrobial Agents

Most of the antimicrobial agents used in commercial textiles are biocides acting in different ways according to their chemical and structural nature and affinity level to certain target sites within microbial cells. Those different modes of action may be [19,20,22]:

- Damage or inhibition of cell wall synthesis, which is critical for the life and survival of bacterial species;
- Inhibition of cell membrane function, which is an important barrier that regulates the intra- and extra-cellular flow of substances, could result in the leakage of vital solutes for the cells’ survival;
- Inhibition of protein synthesis, which is the basis of cell enzymes and structures, consequently leading to the death of the organism or the inhibition of its growth and multiplication;
- Inhibition of nucleic acid synthesis (DNA and RNA) due to the binding of some antimicrobial agents to components involved in the process of DNA or RNA synthesis. This inhibition interferes with normal cellular processes, compromising microbes’ multiplication and survival;
- Inhibition of other metabolic processes, for instance the disruption of the folic acid pathway, which is essential for bacteria to produce precursors important for DNA synthesis.

In Table 1 is presented a summary of the main antimicrobial agents used in textiles, their chemical structure, action modes and the main fibers with which they have been used.

#### 2.1.1. Quaternary Ammonium Compounds (QACs)

These cationic agents, which carry a positive charge at the N atom in solution, are usually attached to an anionic fiber surface by ionic interaction [7,12]. QACs (R_4_N^+^X^−^) represent a large group of 191 compounds, and conventionally, the term QAC refers to a subgroup of linear alkyl ammonium compounds composed of a hydrophobic alkyl chain and a hydrophilic counterpart. In the textile industry, the compounds containing long alkyl chains (12–18 carbon atoms) are the most used, mainly for cotton, polyester, nylon and wool [7,9,23]. The antimicrobial action of these compounds depends on the alkyl chain length, the presence of the perfluorinated group and the cationic ammonium group’s number in the molecule [12].

These compounds are active against a wide range of microorganisms, such as Gram-positive and Gram-negative bacteria, fungi and certain types of viruses [12,24]. Yao *et al.*, for instance, produced poly(d,l-lactide) (PDLLA) fibrous membranes, the surface of which was modified with quaternary ammonium moieties, presenting an antibacterial efficiency of about 99.999% against Gram-positive (*Staphylococcus aureus*) and Gram-negative (*Escherichia coli*) bacteria. It was discussed by the authors that the antibacterial action was based on the interaction of surface positive charges and cell membrane negative charges, resulting in the loss of membrane permeability and cell leakage [25]. In fact, the antimicrobial action is triggered by attractive interactions between the cationic ammonium group and the negatively-charged cell membrane of the microbe. Once in contact with cells, they may present different modes of antimicrobial action, including the damage of cell membranes, the denaturation of proteins and the inhibition of DNA production avoiding multiplication [7,26].

Despite the effectiveness of QACs, they present a disadvantage, namely the leaching from textile due to the lack of physical bonding, resulting in a fast concentration decrease in the textile. In case of Acrilan^®^ and Orlon^®^ commercial polyester fibers, for instance, that contain carboxylic or sulfonate groups, QACs can be directly exhausted under near boiling conditions when washed [27,28]. Moreover, there are also QAC based commercial products in the form of isolated active agents, as presented in Table 2, such as BIOGUARD^®^ (Hamilton, New Zealand), Sanigard KC and Sanitized^®^ (Burgdorf, Switzerland) [12].

#### 2.1.2. Triclosan

Triclosan (2,4,4′-trichloro-2′hydroxydiphenyl ether) (C_12_H_7_Cl_3_O_2_) is an odorless synthetic chlorinated bisphenol not ionized in solutions, unlike most cationic biocides, improving its durability to laundering. It has a wide range of action against Gram-negative and Gram-positive bacteria, but it also presents some antifungal and antiviral properties [7,29,30,47]. This biocide agent mainly acts by blocking lipid biosynthesis, such as phospholipids, lipopolysaccharides and lipoproteins, affecting the integrity of cell membranes [29,30].

Over the last 30 years, this agent has become the most efficient and widely-used bisphenol, namely in many consumer and professional healthcare products, including soaps, lotions and creams, toothpastes, mouthwashes, underarm deodorants and also incorporated into textile fabrics and plastics [7,29]. Triclosan is mainly used in association with polyester, nylon, polypropylene, cellulose acetate and acrylic fibers. There are several products available on the market based on triclosan, as described in Table 1 and Table 3, either as an isolated agent for a finishing option or to incorporate in fibers, such as Microban^®^ (Cannock, United Kingdom) and Irgaguard^®^ (Ludwigshafen, Germany) 1000, or even already incorporated in fiber or fabric form, such as BiofresH™ (Salem, MA, USA) and Silfresh^®^ (Magenta, Italy) [7,12,48].

However, the recent widespread use of triclosan in non-healthcare settings is a great concern due to the potential for selective pressure for certain antibiotic cross-resistant strains, generating bacterial resistance to triclosan [30,47,49]. Moreover, the reported photochemical conversion of triclosan to 2,8-dichlorodibenzo-p-dioxin in aqueous solutions is another great concern due to its toxicity [50,51].

#### 2.1.3. Metals and Metallic Salts

Non-essential metals can be extremely toxic to most microbes at exceptionally low concentrations, either in the free state or in compounds. Due to their biocidal activity, different metal, oxide or salt compounds, mostly based on silver, but also on copper, zinc and cobalt, have been widely used as antimicrobial agents in textiles for agriculture, healthcare and other industries. Often, metals are used in the form of salt-based additives due to the highly expensive metal form [6,7,9]. Their biocidal effect, depending on the metal, can be triggered by the metal reduction potential and/or by the metal donor atom selectivity and/or speciation (the formation of new species in the course of evolution). Therefore, redox-active essential metals can act as catalytic cofactors in a wide range of cell enzymes, either generating or catalyzing reactive oxygen species, which can induce an oxidative stress, damaging cellular proteins, lipids and DNA. In general, metal ions bind to some donor ligands, such as O, N and S, by strong and selective interactions. Thus, external metal ions or their complexes can replace the original metals present in biomolecules, such as in Fe-S clusters, leading to cellular dysfunction [31,32].

The antimicrobial properties of silver particles have been exploited for a long time in general textiles, but mostly in some biomedical textiles, its broad-spectrum action being particularly significant in polymicrobial colonization associated with hospital-acquired infections [6,7]. Silver has revealed a bactericidal activity against a wide range of Gram-positive and Gram-negative bacteria, namely *Pseudomonas aeruginosa*, *S. aureus*, *Staphylococcus epidermidis*, *E. coli* and *Klebsiella pneumonia* [52]. Market data derived from manufacturer surveys in Europe indicated that silver salts are the most widely-used form of silver in textiles, accounting for 79%, while metallic silver and silver ion exchangers are responsible for 13% and 8% of the silver used in textiles, respectively [6,7,9]. Additionally, silver-containing products are also interesting materials for wound repair textile membranes. Metallic silver reacts with moisture on the skin surface or with wound fluids, then silver ions are released and damage the bacterial RNA and DNA, inhibiting their replication. In addition to the antimicrobial action, textile products with a sustained silver release may also manage wound exudates and odor. However, some concerns have been expressed about the development of bacterial resistance to silver-based products [6,13].

Metal nanoparticles are actually presented as an interesting approach, as they present a higher surface area and can dissolve faster in a given solution when compared to larger particles, releasing therefore a higher amount of metal ions and presenting a stronger antimicrobial effect. Moreover, another advantage is that they are easily embedded into fibers’ polymeric matrices [12,32]. Besides silver nanoparticles, also CuO, ZnO and TiO_2_ nanoparticles have been developed, for instance using the sol-gel method, to be associated with textiles [32,53]. ZnO has been reported as presenting a better effect on microorganisms than other metal oxides, such as SiO_2_, MgO or TiO_2_. In addition, the relation between the antibacterial activity versus the ZnO particles size, between 212 and 12 nm, was studied. It was observed that the antibacterial activity is inversely proportional to the nanoparticles’ size [54,55]. Farouk *et al.* have developed a ZnO nanoparticle-chitosan composite to coat cotton fabrics in order to conjugate the antimicrobial power of ZnO and chitosan and even to overcome the lack of strong chemical bonding with textile substrates presented by chitosan. The ZnO nanoparticle-chitosan composite with chitosan of low molecular weight presented a better antibacterial activity. The authors explained this phenomenon by the probably better movement of the nanoparticles to the medium due to a better movement of the polymeric chains in solution when the molecular weight of the composite is lower [55].

In the last few years, in spite of all the cost, environmental and technical challenges related to producing some metal-treated textiles on a commercial scale, significant efforts have been made in industry to develop new efficient antimicrobial textiles. Silver is now used in a large number of commercial antimicrobial products, either as an isolated form, for fiber finishing or incorporation, or even already in fiber or fabric form, as presented in Table 1 and Table 3 [6,7]. As an agent, for instance, there are the products Ultra-Fresh^®^ and Silpure^®^, in which silver is in the form of ultra-fine metallic particles, and it may be applied to fabrics at the finishing stage. Recently, silver nanoparticles, under the trade name SmartSilver^®^, were developed to be applied as a finishing to wool, maintaining its handle and dyeability [7,56]. MicroFresh^®^ and SoleFresh^®^, for instance, are textile yarns based on polyester and nylon polymeric matrix, respectively, with incorporated silver, as well the Bioactive^®^ polyester fibers [7]. Another available product is Silvadur™, which when applied forms an insoluble, interpenetrating polymer network with silver ions on textile fabric surface, allowing a highly durable antimicrobial action up to 50 washings [57].

#### 2.1.4. Chitosan

Chitosan is a natural and hydrophilic copolymer, which results from the deacetylation of chitin extracted from the exoskeleton of crustaceans, like crabs and shrimps, and even from the cell walls of several fungi. It is composed of two monomeric units, d-glucosamine and *N*-acetyl-d-glucosamine, linked by a β(1–4)-glycosidic bond [12,57]. This linear polysaccharide has been widely studied for textiles finishing, mostly for medical applications, due to its biocompatibility, non-toxicity, non-carcinogenicity and antimicrobial activity [13,58,59]. It is mostly used to finish cotton, polyester and wool fibers, and it has presented an antimicrobial activity against a wide spectrum of microorganisms, including fungi, algae and some bacteria [7,33,60,61].

The antimicrobial action mode of chitosan is influenced by intrinsic factors and environmental conditions, namely by the chitosan molecular weight (Mw) and polymerization degree, its deacetylation degree, the pH of the medium and the microorganism type [7,12,33,60]. However, it is generally accepted that the first step is always based on the interaction between the primary amine group’s positive charges and the negative charges on the microbes’ surface. Then, in the case of low Mw water-soluble chitosan, it can penetrate the cell wall, combine with DNA and inhibit the synthesis of mRNA, preventing protein synthesis [12,33]. In the case of high Mw water-soluble chitosan, which presents a higher density of positive charges, the interaction may change the cell membrane, increasing its permeability, which may cause the leakage of some intracellular substances, or it may form an impermeable layer around the cell, blocking the transport of essential solutes into the cell [7,33].

In order to develop even more efficient and durable antimicrobial treatments for textiles, complexes based on chitosan and other more efficient biocidal agents have been studied. Wang *et al.* produced chitosan-metal complexes with bivalent metal ions, including Cu(II), Zn(II) and Fe(II)*.* The complexes showed a wide antimicrobial activity against Gram-positive bacteria (*S. aureus* and *S. epidermidis*), two Gram-negative bacteria (*E. coli* and *P. aeruginosa*) and two fungi (*Candida albicans* and *Candida parapsilosis*). The antimicrobial effect of the complexes was much higher than in the case of free chitosan or metal salts. The authors concluded that the phenomenon was due to the stronger positive charge after complexation [59]. Zhou *et al.* produced nanocapsules based on antibacterial polypeptide-grafted chitosan with an excellent antibacterial efficacy against both Gram-positive and Gram-negative bacteria [62].

The main disadvantages of chitosan as an antimicrobial agent for textiles, either as a finishing or to be incorporated in synthetic fibers, is its poor handling and pH and temperature activity dependence. As chitosan is only soluble in an acidic environment and it becomes polycationic when the pH is below the molecule’s pKa (6.3–6.5), a stronger antibacterial activity has been reported for acidic conditions [63]. Moreover, during storage and depending on temperature, some specific chitosan properties may alter, such as the viscosity and Mw, which consequently affects the biocide effectiveness [64]. However, there is already a commercial product based on this natural polymer to be used as a finishing, the Eosy^®^ product, as presented in Table 2. Moreover, a composite fiber of chitosan and viscose, named Crabyon^®^, presented in Table 4, is commercially available, presenting a durable antimicrobial efficacy [7,12].

#### 2.1.5. Natural-Based Antimicrobial Agents

Due to the emergence of the antibiotic resistance of pathogenic bacteria, antimicrobial compounds extracted from herbs and plants have also been extensively studied as an alternative therapeutic strategy to combat microbial growth in textiles [15,16,65]. Several plant-based compounds with a wide activity spectrum against different fungal and bacterial pathogens have been identified, as presented in Table 3, and are commercially available [66,67,68]. The main advantage of using these natural compounds for antimicrobial purposes is that they do not exhibit the side effects often associated with synthetic chemicals. Until now, no reports of antimicrobial resistance to these natural chemicals have been published. Despite the lack of research about the mechanistic basis of their antimicrobial action, it is supposed that the microbial resistance is prevented probably by their multiple action mechanisms, avoiding the selection of resistant bacteria strains [15,16,66]. Moreover, these substances may present an efficient antimicrobial effect, with safety, easy availability, nontoxicity to skin and being environmentally-friendly [15,16].

In addition to these compounds, natural defensive amino acids and peptides, which are found in every living organism, have also been considered as promising candidates for antimicrobial textile applications. Those antimicrobial peptides (AMPs) are a large number of small proteins that present a broad spectrum of antimicrobial activity against various microorganisms, including both Gram-positive and Gram-negative bacteria [69,70]. Many different AMPs from various families have been discovered in non-vertebrates and vertebrates, and they are characterized by their small size (12–50 amino acids), the arginine and lysine residues responsible for their positive charge, and an amphipathic structure that interacts with microbial membranes. They are usually classified depending on their size, conformational structure or predominant amino acid structure. Several studies have proposed different hypotheses describing the mechanism of AMPs’ action, namely they may affect several internal cellular processes from macromolecular synthesis (*i.e.*, RNA, DNA synthesis) to the loss of ATP from actively-respiring cells [69,70,71]. Many peptides are already used in medicine, such as daptomycin (Cubicin^®^, Cubist Pharmaceuticals), pexiganan and psoriazyna. Besides that, there are many new antimicrobial peptides displaying interesting properties that are currently under development, such as plectasin NZ2114, which reveals potent bactericidal activity against Gram-positive pathogens [69,70,72]. Another efficient AMP is l-cysteine, which has been successfully used to promote the biofunctionalization of wool and polyamide, granting to those fibers a durable antimicrobial effect [73].

#### 2.1.6. Poly(Hexamethylene Biguanide) (PHMB)

Polybiguanides are polycationic amines composed of cationic biguanide repeat units separated by aliphatic chains. One of the most used biguanide-based polymers is poly(hexamethylene biguanide) (PHMB) ((C_8_H_17_N_5_)*_n_*), which has a hydrophobic backbone in which the cationic biguanide groups are interspersed between hydrophobic hexamethylene groups [34,35]. Both the cationic and hydrophobic features of PHMB suggest that it interacts with microbial cell membranes by electrostatic and hydrophobic interactions. Those interactions with the membrane phospholipids result in cell membrane disruption and lethal leakage of cytoplasmic materials, and its activity increases on a weight basis with increasing levels of polymerization [7,34,35].

The commercial current applications of PHMB are mainly in health (mouthwashes and wound dressings), clothing, household and water treatment textiles [7,35]. Some PHMB-based products have already appeared on the market, but only as a finishing form, as presented in Table 2, such as Biozac ZS and Reputex^®^ (with an average of 16 biguanide units), the respective textile products of which are found under the trademark Purista^®^ (Table 4). Due to the longer length, Reputex^®^ presents more cationic sites, promoting a stronger binding to the fiber surface and also a higher biocidal activity [7,12,34].

#### 2.1.7. Regenerable N-Halamines

N-halamines are heterocyclic organic compounds, which may contain one or two covalent bonds between nitrogen and a halogen (N–X), which is usually chlorine (N–Cl), but it can also be bromine or iodine. Those covalent bonds can be formed by the chlorination of amine (RR′−NX), amide (−C(O)−NX−R) or imide (−C(O)−NX−C(O)−) groups in dilute sodium hypochlorite, presenting different stability levels, the trend of which is determined by their structure following the order: imide < amide < amine. However, the antimicrobial effectiveness of these three types of N-halamines follows the inverse order: imide > amide > amine [7,8,12]. N-halamines present a biocide action against a broad spectrum of bacteria, fungi and viruses, which is triggered by the electrophilic substitution of Cl with H in the presence of water. The free Cl cations bind to the acceptor regions on microorganisms, precluding the cell enzymatic and metabolic processes, causing the consequent microorganism destruction [8,12]. However, depending on the purpose, the stability and effectiveness levels shall be taken into account when choosing the halamine type to use. If it is intended to rapidly destruct the microorganisms, the imide N-halamine compounds must be used, but if it is intended to sustain the antimicrobial properties of the textile for the long term, the amine N-halamine will be the right choice [8,85].

In addition to the wide range of action, the long-term stability and the low cost, the main advantage of these antimicrobial agents is the possibility of constantly recycling their antimicrobial effect by recharging the inactive substance by simply reacting them with Cl or Br donor compounds, such as sodium hypochlorite, sodium hypobromite, trichloroisocyanuric acid or sodium dichlorocyanurate, using a bleaching solution during laundering [8,85]. As a disadvantage, some studies have reported that the textiles’ treatment with N-halamine may result in a substantial amount of adsorbed Cl or even other halogens on the fiber surface. Those residues may produce an unpleasant odor or even discolor fabrics, which is a concerning disadvantage to the textile industry [8,12].

These compounds have been covalently applied to different types of textile fibers, including chitosan, cellulose, cotton, polyamide and polyester fibers [8,86,87]. N-halamine polymers can be prepared by three different approaches, namely by a polymerization approach, by electrogeneration or just by chemical grafting to the polymeric surfaces, as discussed in the next section [85]. Furthermore, as antibacterial materials with a larger surface area present more N-halamine functional sites to contact with bacteria, presenting higher biocidal action, some research has been done to develop nano-N-halamine composites [88].

### 2.2. Antimicrobial Textiles by Finishing Treatments with Antimicrobial Agents

As already referred, antimicrobial polymers can be prepared either by embedding an antimicrobial agent into the polymer bulk during their processing or by applying a surface coating or modification as a chemical or physical finishing treatment. Over the past decade, several surface grafting techniques have been studied; however, the method used strongly depends on if the textile fiber is natural or synthetic and also on its physico-chemical features. Different techniques have been used to achieve textiles surface grafting, such as: (1) chemical grafting; (2) plasma-induced grafting using either radiofrequency or microwave plasma; (3) radiation-induced grafting, which uses high-energy radiation (e.g., γ-Co60 rays); and (4) light-induced grafting using a source of ultraviolet radiation [24,52,89].

Regarding chemical grafting polymerization, conventional finishing techniques applied to textiles, such as dyeing, stain repellence, flame retardance or antibacterial treatments, generally use wet chemical process steps. In wet chemical surface modification, the textile surface is treated with liquid reagents, which penetrate into the textile fabric in order to create reactive functional groups on the surface or to initiate copolymerization reactions with different antimicrobial monomers. Therefore, the surface functionalization degree is not repeatable between polymers with different molecular weight and crystallinity levels. This process may also lead to the generation of hazardous chemical wastes and can result in textile surface etching [6,89]. As an alternative, plasma treatment provides a clean technology for polymers’ surface modification without affecting their bulk properties. The free radicals and electrons created in the plasma enable the surface activation by chemical bonds breaking to create reactive sites, functional groups or for later grafting of antimicrobial chemical moieties [6,18,52,89]. Davis *et al.* for instance, treated a cotton/PET blend fabric with a water-repellent treatment through activating the surface with plasma, depositing vaporized fluorocarbon-based monomers and then graft polymerizing the monomer with a second plasma exposure. The fabrics were then further treated with an antimicrobial agent, namely a quaternary ammonium salt. Plasma treatment was used to induce free radical chain polymerization of the agent, resulting in a graft polymerized network on the fabric. It was shown that the water repellent treatment was efficient to obtain a high hydrophobic fabric with high durability to laundering. The results of the antimicrobial tests showed an activity reduction of both Gram-positive and Gram-negative bacteria by more than 99.994%, proving that the antimicrobial agent can act effectively on the water repellent-treated fabric [90]. Moreover, cotton fabrics have been preprocessed with plasma in order to increase the surface roughness to allow the loading of higher concentrations of silver and zinc oxide, which are used as catalyst to promote the reaction between halogenated phenoxy compounds with Microban [91].

In case of QACs, in order to increase their durability, promoting a stronger bond between QACs and the fibers’ surface, some polymerizable QACs with acrylate or methacrylate groups have been synthesized. Those QACs monomers may be polymerized into a bulk polymer network with a polycationic structure, including side QAS groups chemically bonded to the main polyacrylate chain. The network fixed bonding to the textile surface allows the QAC groups to act as a biobarrier and against microorganisms by contact (non-leaching action), increasing the agent durability under washing [92,93]. Another studied approach is to fix QACs on fibers by sol-gel technology, enabling the formation of a nanocomposite polymeric network with an organic-inorganic hybrid structure, allowing a more controlled agent release. The sol-gel technique allows one to tailor certain properties and to combine different properties in a single coating step [89,94]. Moreover, according to Sun *et al.*, for some synthetic fibers that contain fewer reactive sites or that are not resistant to chemical modification, dye molecules may be used as bridges to bind QACs to the fiber surface [95]. This method has also been widely used to improve the binding of PHMB to cotton fibers [96,97]. However, despite that the ionic interaction between the dye molecules and the antimicrobial agent may promote a more durable finishing, the strong ionic bonding may decrease the release of free PHMB or QACs and consequently the antimicrobial efficiency [7].

When using metals as the antimicrobial agent, the treatment of natural fibers can only be undertaken at the finishing stage, and various strategies have been studied to enhance the uptake and durability. Cotton, for instance, has been pre-treated with succinic acid anhydride, which acted as a ligand for metal ions (Ag^+^ and Cu^2+^) of metallic salts to provide very effective antibacterial activity [6,32,53]. Plasma pre-treatment has also been used to create active groups on synthetic and natural polymers’ surface to be combined with TiO_2_ and SiO_2_ nanoparticles. Won *et al.* have even produced nanoparticles in the polymeric matrix via reducing metallic salts under irradiation with UV light [53]. Moreover, some authors have proposed the association of the nanoparticles with a polymeric matrix, such as silver nanoparticles associated with polysiloxane, to improve the durability of the antimicrobial effect. The polymeric matrix on the fabric surface, with embedded nanoparticles, works as a controlled delivery system of ions, promoting the long-term antimicrobial activity, especially against laundering, as happens with the commercial product Silvadur™ [32,53].

In the case of PHMB, which is water soluble, the conventional processes, such as padding and exhaustion, are suitable application methods. Reputex^®^, for instance, may be applied to cotton or its blends using exhaust or pad-dry-cure processes, and more recently to polyester or nylon fabrics with the name Purista^®^ [7]. In those processes, the cationic biguanide groups are involved in binding the polymer to the textile fabric surface by electrostatic interactions with negatively-charged groups, such as carboxylic groups in cellulose fibers, being also used for cotton, polyester and nylon fibers [7,35,98]. The carboxylic groups when using a cellulosic fabric are formed through oxidation of glucose rings during pre-treatment processes, such as bleaching and mercerizing [12].

N-halamine polymers can be prepared just by grafting N-halamine monomers onto the polymer backbones or by modifying the polymer units to form N-halamine derivatives [85]. In the case of cellulose, for instance, N-halamide monomers with an incorporated vinyl reactive group have been developed to polymerize on fibers’ surface [8,12]. Moreover, Badrossamay *et al.* have prepared biocidal polypropylene by incorporating onto its backbone several acyclic N-halamine precursors, such as acrylamide, methacrylamide, N-tert-butylacrylamide and N-tert-butylmethacrylamide [99]. Another approach is to promote the polymerization of N-halamine monomers or monomers bearing N−H function by themselves or with other monomers to generate biocidal homopolymers or heteropolymers [85]. Ahmed *et al.*, for instance, synthesized a novel N-halamine polyurethane by copolymerizing a heterocyclic ring-based monomer with either tolylene-2,6-diisocyanate or toluene-2,4-diisocyanate, resulting in halogenated polyurethanes, namely chlorinated, brominated or iodinated [88]. Ren *et al.* developed an antimicrobial coating based on a N-halamine monomer on cotton fibers using the admicellar polymerization process. This is a very recent process used for textiles’ surface modification, yet its industrial acceptance has not been reported so far [86,100].

Furthermore, textile surfaces can also be coated by the layer-by-layer assembly technique (LbL), which is mainly conducted through electrostatic interactions. The assembly begins with the adsorption of a charged material onto a substrate with the opposite charge, which leads to charge neutralization and then charge reversal. Further layers are then deposited by the sequential alternate adsorption of the oppositely-charged materials onto the substrate until the desired multilayers are reached [85,89]. Recently, Cerkez *et al.* used this technique to grant antimicrobial properties to cotton fabrics, which when bleached are inherently negatively charged, allowing the deposition of a multilayer thin film by alternative exposure to polyelectrolyte solutions based on two N-halamine copolymers [101]. This technique has also been successfully used to promote a very stable immobilization of AMPs, as proposed by Zhong *et al.*, who used oppositely-charged polypeptide to make 10-bilayer polyelectrolyte multilayer films by electrostatic LbL assembly [70]. Since the charged copolymers are very soluble in water and due to the simplicity and low cost, because no special or complicated instruments are needed, this coating technique may have industrial potential for antimicrobial coating of textiles [85,89,101].

Another possible process is the enzymatic surface modification of textiles, which increases the hydrophilicity and removes components from the surface, even allowing the introduction of functional groups on the surface. Gaffar Hossain *et al.*, for instance, developed a process to grant antibacterial properties to wool using an enzymatic process. This technology increases the reaction rate of (bio)chemical reactions without these being consumed themselves, just like chemical catalysts; however, enzymes are often much more effective at enhancing reaction rates than chemical catalysts. The weaknesses of enzyme surface modification techniques are related to the slow diffusion of enzymes compared to regular chemicals, the limited temperature stability and the sometimes low reaction rates on synthetic materials [89].

It should be mentioned that some studies have reported that just by modifying the surface properties of a material, such as surface free energy, polarity or topography, it is possible to decrease the bacterial adhesion to the surface, during the initial stage of the biofilm formation process, without using chemical antimicrobial agents. Those modifications, performed for instance by applying wet chemistry through reaction with chemical reagents or by plasma treatment, may create new functional groups and/or change the surface roughness. However, the modifications promoted by plasma treatment present a weak stability over time, as the polymeric surfaces tend to return to their original chemical state [52,102,103].

### 2.3. Antimicrobial Textiles by the Incorporation of Antimicrobial Agents in Fibers

Another approach to develop textiles with an antimicrobial ability is by the incorporation of the antimicrobial agents into the polymeric matrix of the textile fibers. Based on this, there are different products already available on the market, as presented in Table 4.

The agents may be incorporated into the polymeric granules prior to the production or just added to the chamber during the extrusion or electrospinning process [6,7,11]. The sub-micron range of fibers produced by electrospinning reveals several advantages, such as a high surface area to volume ratio, adjustable porosity and the ability to manipulate the nanofiber composition to obtain the desired properties [104]. Duan *et al.*, for instance, have produced antibacterial electrospinning nanofibers based on poly(ε-caprolactone) (PCL) with nanoparticles of silver-loaded zirconium phosphate (nano-AgZr) with high potential for wound dressing usage [105]. In addition, Ignatova *et al.* studied the electrospinning of poly-vinyl-pyrrolidone iodine complex and poly-ethylene oxide/poly-vinyl-pyrrolidone iodine complex, revealing also potential results to be used in antimicrobial wound dressing materials [105]. Further, ZnO nanoparticles may be electrospun with polyurethane fibers, which are then deposited on a cotton textile fabric surface [106].

The direct addition of the biocide agent into the polymers has received considerable attention, especially when using thermoplastic matrices. The main advantage of this method is that it may be easily implemented in the standard and large-scale processing units already designed to prepare particulate-filled polymer composites, which are extensively used in the textile industry. Besides that, when using a low agent content, the resulting mechanical properties of the modified fibers are similar to the unmodified ones. The main disadvantage of this method is the lower antimicrobial power due to the restricted diffusion of the antimicrobial agent molecules through the polymeric matrix. Most of the incorporated agent gets trapped in the matrix, not being available to interact with microorganisms and to perform its biocide or bacteriostatic function. However, the agents’ incorporation may provide better durability, as the agent is physically withheld in the polymeric matrix, promoting a slower release during use [6,11,103].

However, this agent’s incorporation on textile fibers may only be used for some synthetic polymers due to the high standard processing temperature used in an extrusion process. In addition, it is also necessary to carefully select the antimicrobial agent regarding its temperature stability. Therefore, metallic particles or even nanoparticles are the most used agent in this method, as they do not present degradation when submitted to the standard processing conditions of thermoplastic polymers, as proven by the number of commercial products based on this presented in Table 4 [6,32,103]. There are also some products available on the market in the form of fibers or fabrics based on natural fibers possessing an intrinsic antimicrobial ability, such as Chitopoly^®^ and Crabyon^®^, which are based on chitosan and cellulose fibers. However, the biocide and bacteriostatic action of these kinds of products is less efficient than the ones that use the most conventional antimicrobial agents presented in this review.

## 3. Environmental and Health Impact of Antimicrobial Agents

Regarding the development of antimicrobial treatments for textiles, the advantages of the chosen antimicrobial agent, such as biocide effectiveness, should outweigh the potential environmental consequences and costs of its usage. As environmental benefits, antimicrobial-treated textiles may present a prolonged useful period of textile wear, the potential for less frequent laundry cycles, lower washing temperatures and a reduction of detergent consumption in each wash cycle. The most desirable agents are the ones that are simultaneously efficient at low dosing levels and that present durable functionality. The environmental impact of the antimicrobial textile, which is a critical global concern, is related to the textile substrate material, antimicrobial compound production, textile treatment processes and also the subsequent use and disposal of the product [9,114].

When antimicrobials are released from textiles, if they are not removed in the waste water treatment, they may end up in the aquatic environment. Several studies have revealed that large portions of silver (including metallic Ag and Ag nanoparticles) and triclosan are effectively removed during waste water treatment, namely 85%–99% and 90%–96%, respectively [9]. In the case of QACs, for instance, due to their positive charge, the cations tend to strongly adsorb to negative surfaces of sludge, soil and sediments. Besides the removal through sedimentation, biodegradation is another possible process that enables the elimination of the compounds in waste water and in the aquatic environment [9,26]. QACs, for instance, are generally referred to as “hard antibacterial agents” as they are poorly metabolized and mainly excreted in a non-metabolized form. The biodegradability of QACs is generally considered as poor; however, considerable variation has been observed depending on the concentration of the substance [26]. Triclosan, for instance, is degradable when subjected to aerobic conditions, but it may be persistent under anaerobic conditions. The triclosan photolysis half-life, which is the main degradation process, has been estimated as about 41 min and the half-life in lake water as about 10 days [9,115]. The triclosan degradation products, such as methyl triclosan, are potentially more toxic than the parent compound, which is a big concern when using this antimicrobial agent [9]. By definition, as mineral-derived materials, silver-based products cannot be considered as biodegradable; however, all silver forms are subject to some transformation processes that may lead to their removal from the aquatic environment. Some studies revealed that nano-Ag may be immobilized by the formation of stable sulfide complexes, resulting in silver sulfide, which is very insoluble and much less toxic and bioavailable than dissolved silver. Therefore, the formation of silver sulfide would significantly decrease the risk of silver-based compounds in the aquatic environment [9,116].

Moreover, the introduction of rigorous ecological legislation is forcing companies to consider not only the issue of industrial waste disposal, but also the possible replacement of conventional processes with new approaches that can provide equal or even higher efficiency and lower environmental impact. The use of plasma technologies for pre-treatments, for instance, is increasingly replacing conventional wet chemical applications, not only for economical considerations, but also for the ecological advantages. The dry plasma treatment does not employ harmful chemical solutions (solvent-free process) and does not produce contaminated water or create mechanical hazards for treated fabrics [117]. Another environment-friendly approach that is becoming increasingly used in textiles is the promotion of specific reactions catalyzed by enzymes. Among the most used enzymes for polymer modifications are, for instance, glycosidases, proteases, lipases and laccase. In order to have wool fibers with an antimicrobial ability, protease, for instance, was used to treat the fibers with antimicrobial natural dyes, and it was demonstrated that the enzyme process enhances the quantity of natural dye exhausted [118,119]. In addition, the ecological treatment of plant fibers with fungi is also an alternative method to the conventional chemical methods. Fungal treatment causes the formation of holes on the fibers’ surface, providing roughness to their surface and increasing the interfacial adhesion between fibers and the surrounding matrix [119]. Furthermore, ultrasound energy and UV radiation have also been exploited as techniques that are non-harmful to the environment to promote the dyeing of textiles, in which dyes may present antimicrobial properties [118].

Besides the “green” technologies, researchers have also studied the usage of “green” modifying antimicrobial agents, such as natural biopolymers extracted from animals or plants, namely chitosan, cyclodextrin, sericin, alginate and others, which are renewable and have the potential to be a key resource in the development of sustainable bioactive textiles [120]. Hu *et al.*, for instance, used a “green” approach to modify cellulose fibers’ surface by using alkali lignin as a functional agent to bind onto the fibers’ surface, then complexed with silver cations and reduced them to atomic silver by growing into nanoparticles [121]. Khurshid *et al.* have developed an eco-friendly natural antimicrobial textile finish for cotton, using extracted Aloe vera gel mixed with an active substance of Neem plants. This hybrid combination presented an effective antibacterial and antifungal activity and even good durability to washing [122].

Microorganisms play an important role in the cycling of elements at a global scale, thus profoundly and directly affecting the environments. In spite of most bacteria being harmless, some may be beneficial to their host, providing nutrients or protection from pathogens and diseases by limiting the ability of more harmful bacteria to colonize. If microorganisms affect their surrounding environment, the environment in turn also engenders evolutionary pressures on the microorganisms. They have a short generation time, lasting from minutes to hours, being able to rapidly respond to changes in their environment. Therefore, as antimicrobial agents are introduced into the environment, microorganisms respond by becoming resistant to these agents [9,123]. The antimicrobial resistance mechanisms result from changes in the cellular physiology and structure of a microorganism due to changes in its usual genetic makeup by acquiring genes from resistant microorganisms in the same niche (acquired resistance) or developing novel ways to prevent the entrance of those agents (intrinsic resistance) [17,123]. In the case of QACs, some studies have reported bacterial resistance to them, either intrinsic or acquired. Intrinsic resistance is demonstrated by Gram-negative bacteria, bacterial spores, mycobacteria and under certain conditions by *Staphylococci.* This is achieved by the reduction of the cell membrane permeability through modifications in phospholipids’ synthesis or by membrane proteins’ linkage [26,49]. In the case of silver compounds, some studies have reported antibacterial resistance against the agent, and the ease with which silver resistance can become selected in some bacteria suggests that there would be a benefit in improved surveillance for silver-resistant isolates in the clinic, along with a better control over silver use, in order to best preserve its clinical utility [7,124,125].

The development of resistance is a normal evolutionary process for microorganisms. Albeit, this phenomenon is strongly accelerated by the selective pressure exerted by widespread use of antibacterial drugs, becoming a great global public health concern of increasing magnitude. Resistant strains are able to propagate and spread [123,126,127]. Therefore, the potential health effects need to be considered in order to evaluate the safety of antimicrobial compounds for animals and humans. The type of action of the antimicrobial agent, by diffusion or contact, its concentration in the textile, the exposure routes and the frequency of use will influence the extent to which humans may be exposed to the textile. Some parameters usually evaluated to assess the risk profile of an antimicrobial agent associated with a textile are the toxicity (acute and chronic), skin sensitization and irritation and the disturbance of skin ecology. For all of the antimicrobial agents presented in this review, there are no conclusions regarding their skin toxicity and effects on human health. In the case of triclosan, its toxicity is a concern, as it is suspected that it may act as an endocrine disrupter and has been observed to be distributed in human tissues. In the case of Si-QAC (silane quaternary ammonium compounds), according to some studies, no severe effect on human health was observed. In the case of metallic compounds, ZnPT (zinc pyrithione), for instance, may present some risks of neurotoxicity [9,26]. Regarding Ag ions, which are non-specific in action as other biocides, they may interact with skin flora, causing the detachment of the cytoplasmic membrane from the cell wall of healthy bacteria, weakening the skin defense barrier [128,129]. Furthermore, chronic contact with Ag can lead to deposition of Ag metal/Ag sulfide particles in skin, inducing its discoloration, known as argyria, or even ocular discoloration, known as argyrosis. These are not life-threatening conditions, but of course, cosmetically undesirable [128].

In order to influence the antimicrobials’ use pattern and the market share of particular compounds, some changes in the applicable regulation should be performed. Thus, the antimicrobial use pattern in textiles would be modulated according to the specific application and need, resulting in a significant reduction of antimicrobials’ flow to the environment. For instance, the textiles industry uses the highest proportion of total used triclosan, while the use of QACs represents the minor proportion of all used QACs. The 210 metric tonnes of used triclosan in textiles can be replaced by either less than two metric tonnes of silver nanoparticles or by 180 metric tonnes of Si-QAC, reaching similar antimicrobial results with a lower environmental impact. Therefore, the regulatory assessment of single antimicrobial substances should be based on a broader perspective and knowledge of antimicrobials, taking into account the risks and benefits of their use compared to alternative antimicrobial substances [9,127].

## 4. Conclusions

In the last few years, the customer’s desire for comfort, hygiene and well-being, concerning odor control and microorganisms protection, has created a large and rapidly increasing market for the expansion of antimicrobial textiles. As discussed in this article, the manufacturing industries have reacted to this demand by launching their brands of antimicrobial products as isolated antimicrobial agents, including organic and inorganic compounds, or in fiber or fabric form. Those agents may be applied at the finishing stage or incorporated into synthetic fibers. In order to ensure a chemical reaction between the agent and the fibers, it is important to use agents with reactive groups, crosslinking agents as intermediates and to pre-treat the textile fibers to increase the concentration of functional groups. The products’ effectiveness and durability depend on the polymer type of the textile fiber, the agent and the finishing or incorporation technique used in production.

Despite the advantages associated with the antimicrobial textiles, the bacterial resistance promoted by the agents, their toxic breakdown products and the consequent risks to human health and environment balance have been a great concern. The long-term benefits and potential problems associated with antimicrobial textiles should be simultaneously considered and closely monitored to avoid the problems overpassing the benefits. Therefore, in the future, more efforts must be done by researchers and manufacturers in order to develop more environment-friendly techniques and to promote the usage of “green” agents, namely natural-based compounds, which have revealed potential antimicrobial activity without harmful effects to the surrounding medium. Another future suggestion is to explore the agents’ micro-encapsulation. This could improve their durability on the textile matrix and simultaneously improve the human and environment safety level.

## Figures and Tables

**Table 1:** Chemical structure and action modes of the main antimicrobial agents, as well as the main fibers in which they are used. QAC, quaternary ammonium compound; PHMB, poly(hexamethylene biguanide).

Biocide	Chemical Structure	Action Modes	Fibers
**QACs**	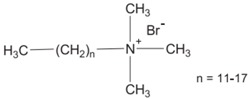 (Example: monoquaternary ammonium salt: alkyltrimethylammonium bromide)	•Damage cell membranes; •Denature proteins; •Inhibit DNA production, avoiding multiplication [7,12,26].	Cotton Polyester Nylon Wool
**Triclosan**	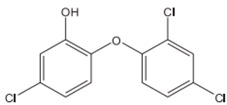	Blocks lipid biosynthesis, affecting the integrity of cell membranes [7,29,30].	Polyester Nylon Polypropylene Cellulose acetate Acrylic
**Metals and metallic salts**	Examples: TiO_2_ and ZnO	Generate reactive oxygen species, damaging cellular proteins, lipids and DNA [31,32].	Cotton Wool Polyester Nylon
**Chitosan**	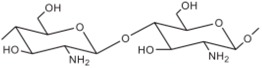	•Low Mw: inhibits synthesis of mRNA, preventing protein synthesis [12,33]; •High Mw: causes leakage of intracellular substances or blocks the transport of essential solutes into the cell [7,33].	Cotton Polyester Wool
**PHMB**	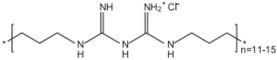	Interacts with membrane phospholipids, resulting in its disruption and the lethal leakage of cytoplasmic materials [7,34,35].	Cotton Polyester Nylon
**N-halamines**	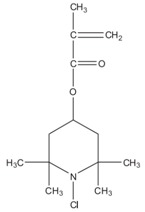 (Example: *N*-chloro-2,2,6,6-tetramethyl-4-piperidinyl methacrylate)	Precludes the cell enzymatic and metabolic processes, causing the consequent microorganism destruction [8,12].	Cotton Polyester Nylon Wool

**Table 2:** Commercially available antimicrobial isolated agents.

Product Name	Company	Description
**agion^®^ [36]**	Sciessent	Additive based on silver and zeolite
**AlphaSan^®^ [37]**	Milliken Chemical	Additive based on silver
**BioGuard^®^ [38]**	AEGIS Microbe Shield™	Finishing agent based on 3-trimethoxysilylpropyldimethyloctadecyl ammonium chloride
**Biozac ZS [12]**	Zschimmer & Schwarz Mohsdorf GmbH & CoKG	Finishing agent based on PHMB
**Cosmocil CQ™ [39]**	Lonza	Additive based on polyaminopropyl biguanide
**Eosy^®^ [12]**	Unitika	Finishing agent based on chitosan
**Irgaguard^®^ 1000 [12]**	BASF (Ciba)	Finishing agent based on triclosan
**Irgasan [12]**	Sigma Aldrich	Finishing agent based on triclosan
**Microban^®^ [40]**	Microban International	Agent based on triclosan
**Reputex™ [41]**	Lonza	Finishing agent based on PHMB
**Sanigard KC [42]**	L.N.Chemical Industries	Finishing agent belonging to the QAC group
**Saniguard Nano-ZN [42]**	L.N.Chemical Industries	Finishing solution based on an aqueous nano-dispersion of zinc oxide
**Sanitized^®^ [43]**	SANITIZED	Finishing agent based on 3-trimethoxysilylpropyldimethyltetradecyl ammonium chloride
**Silpure^®^ [44]**	Thomson Research Associates	Finishing agent based on fine silver particles
**Silvadur™ [45]**	The Dow Chemical Company	Interpenetrating polymer network with silver ions
**SmartSilver^®^ [12]**	Nanohorizon Inc.	Agent based on silver nanoparticles
**Silvérion 2400 [46]**	PURE Bioscience, Inc.	Agent based on a stabilized silver complex

**Table 3:** Different plant-based compounds’ chemical structure and respective antimicrobial spectrum.

Plant-Based Antimicrobial Agents	Chemical Structure	Antimicrobial Spectrum
**Alkaloids**		
**Terpenoids [74,75]**	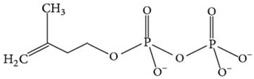	*- Staphylococcus aureus* *- Pseudomonas aeruginosa* *- Vibrio cholera*
**Lectins and Polypeptides [68,76]**	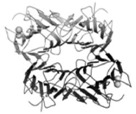	*- Staphylococcus aureus* *- Bacillus subtilis* *- Escherichia coli* *- Pseudomonas aeruginosa* *- Candida albicans*
**Phenolics and polyphenols**		
**Flavonoids [77,78,79]**	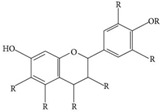	*- Klebsiella pneumonia* *- Salmonella enterica* *- Pseudomonas aeruginosa* *- Staphylococcus aureus* *- Escherichia coli* *- Acinetobacter baumannii*
**Quinones [80,81]**	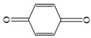	*- Staphylococcus aureus* *- Bacillus subtilis* *- Pseudomonas aeruginosa*
**Tannins [82,83]**	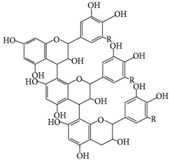	*- Bacillus cereus* *- Listeria monocytogenes* *- Staphylococcus aureus* *- Salmonella enterica*
**Coumarins [66,67,84]**	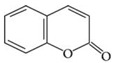	*- Listeria monocytogenes* *- Staphylococcus aureus* *- Escherichia coli* *- Vibrio parahaemolyticus*

**Table 4:** Commercially available antimicrobial textile fibers or structures.

Product Name	Company	Description
**ACTICOAT™ [107]**	Smith & Nephew	Textile structure composed of 3 layers: 2 layers of polyethylene mesh coated with high density nanocrystalline silver; 1 layer of rayon and polyester
**Amicor/Amicor Plus [12]**	Acordis, Ltd.	Acrylic fibers containing triclosan or a combination of triclosan and tolnaftate
**Bactekiller^®^ [12]**	Fuji Chemical Industries, Ltd.	Fibers containing metal ions
**Bactershield^®^ [108]**	Sinterama	Polyester yarn containing a bacteriostatic agent
**Bioactive^®^ [109]**	Trevira	Polyester fibers containing silver
**BiofresH™ [12]**	Sterling Fibers, Inc.	Acrylic fibers containing triclosan
**Chitopoly^®^ [12]**	Fuji-Spinning	Fiber made by kneading chitosan into polynosic fiber
**Crabyon^®^ [110]**	SWICOFIL AG	Composite fiber of chitin/chitosan and cellulose viscose
**FeelFresh^®^ [111]**	Toyobo	Acrylic fibers endowed with antibacterial metal ions
**Kendall™ [112]**	Medtronic	Textile foam dressing containing PHMBs
**Microfresh^®^ [7]**	O’Mara, Inc.	Polyester yarns containing silver particles
**Rhovyl’As^®^ [12]**	Rhovyl	Fibers containing triclosan
**SeaCell^®^*active* [113]**	Smartfiber AG	SeaCell fibers (based on cellulose) enriched with silver ions
**Silfresh^®^ [7]**	Novaceta	Cellulose acetate yarn containing triclosan
**SoleFresh^®^ [7]**	O’Mara, Inc.	Polyester yarns containing silver particles
**Thunderon^®^ [12]**	Nihon Sanmo Dyeing Company, Ltd.	Acrylic fibers containing copper ions

## References

[1] FAO (Food and Agriculture Organization of the United Nations) and ICAC (Cotton Advisory Committee) (2011). A Summary of the World Apparel Fiber Consumption Survey 2005–2008.

[2] Textile World Man-Made Fibers Continue to Grow. http://www.textileworld.com/textile-world/fiber-world/2015/02/man-made-fibers-continue-to-grow/.

[3] Collier B.J., Tortora P.G. (2001). Understanding Textiles.

[4] Hollen N.R., Saddler J., Langford A.L. (1979). Textiles.

[5] Singleton J. (2013). The World Textile Industry.

[6] Shahidi S., Wiener J. (2012). Antimicrobial Agents—Chapter 19: Antibacterial Agents in Textile Industry.

[7] Gao Y., Cranston R. (2008). Recent advances in antimicrobial treatments of textiles. Text. Res. J..

[8] Zanoaga M., Tanasa F. (2014). Antimicrobial reagents as functional finishing for textiles intended for biomedical applications. I. Synthetic organic compounds. Chem. J. Mold..

[9] Windler L., Height M., Nowack B. (2013). Comparative evaluation of antimicrobials for textile applications. Environ. Int..

[10] Intelligence T. (2014). Demand for Antimicrobial Fibres, Textiles and Apparel Is Set for Strong Growth Performance Apparel Markets. http://www.innovationintextiles.com/demand-for-antimicrobial-fibres-textiles-and-apparel-is-set-for-strong-growth/.

[11] Bshena O., Heunis T.D., Dicks L.M., Klumperman B. (2011). Antimicrobial fibers: Therapeutic possibilities and recent advances. Future Med. Chem..

[12] Simoncic B., Tomsic B. (2010). Structures of novel antimicrobial agents for textiles—A review. Text. Res. J..

[13] Bartels V. (2011). Handbook of Medical Textiles.

[14] Weinstein R.A. (2001). Controlling antimicrobial resistance in hospitals: Infection control and use of antibiotics. Emerg. Infect. Dis..

[15] Upadhyay A., Upadhyaya I., Kollanoor-Johny A., Venkitanarayanan K. (2014). Combating pathogenic microorganisms using plant-derived antimicrobials: A minireview of the mechanistic basis. BioMed Res. Int..

[16] Savoia D. (2012). Plant-derived antimicrobial compounds: Alternatives to antibiotics. Future Microbiol..

[17] Nwosu V.C. (2001). Antibiotic resistance with particular reference to soil microorganisms. Res. Microbiol..

[18] Shishoo R. (2007). Plasma Technologies for Textile.

[19] Michigan State University (2011). Antimicrobial Resistance Learning Site.

[20] Glazer A.N., Nikaido H. (2007). Microbial Biotechnology: Fundamentals of Applied Microbiology.

[21] Rahman M.A., Ahsan T., Islam S. (2010). Antibacterial and antifungal properties of the methanol extract from the stem of argyreia argentea. Bangladesh J. Pharmacol..

[22] Russell A.D., Furr R., Maillard J.-Y. (1997). Microbial susceptibility and resistance to biocides. ASM News.

[23] Kegley S., Hill B., Orme S., Choi A. (2010). Pan Pesticide Database, Pesticide Action Network, North America (San Francisco, CA, 2010).

[24] Yao C., Li X., Neoh K., Shi Z., Kang E. (2008). Surface modification and antibacterial activity of electrospun polyurethane fibrous membranes with quaternary ammonium moieties. J. Membr. Sci..

[25] Yao C., Neoh K., Shi Z.-L., Kang E. (2010). Antibacterial poly(d,l-lactide)(pdlla) fibrous membranes modified with quaternary ammonium moieties. Chin. J. Polym. Sci..

[26] Hegstad K., Langsrud S., Lunestad B.T., Scheie A.A., Sunde M., Yazdankhah S.P. (2010). Does the wide use of quaternary ammonium compounds enhance the selection and spread of antimicrobial resistance and thus threaten our health?. Microb. Drug Resist..

[27] Cai Z., Sun G. (2004). Antimicrobial finishing of acrilan fabrics with cetylpyridinium chloride. J. Appl. Polym. Sci..

[28] Cai Z., Sun G. (2005). Antimicrobial finishing of acrilan fabrics with cetylpyridinium chloride: Affected properties and structures. J. Appl. Polym. Sci..

[29] Orhan M., Kut D., Gunesoglu C. (2007). Use of triclosan as antibacterial agent in textiles. Indian J. Fibre Text. Res..

[30] Yazdankhah S.P., Scheie A.A., Høiby E.A., Lunestad B.-T., Heir E., Fotland T.Ø., Naterstad K., Kruse H. (2006). Triclosan and antimicrobial resistance in bacteria: An overview. Microb. Drug Resist..

[31] Xu F.F., Imlay J.A. (2012). Silver (i), mercury (ii), cadmium (ii), and zinc (ii) target exposed enzymic iron-sulfur clusters when they toxify *Escherichia coli*. Appl. Environ. Microbiol..

[32] Palza H. (2015). Antimicrobial polymers with metal nanoparticles. Int. J. Mol. Sci..

[33] Kong M., Chen X.G., Xing K., Park H.J. (2010). Antimicrobial properties of chitosan and mode of action: A state of the art review. Int. J. Food Microbiol..

[34] Varesano A., Vineis C., Aluigi A., Rombaldoni F. (2011). Antimicrobial polymers for textile products. Sci. Microb. Pathog. Commun. Curr. Res. Technol. Adv..

[35] Chadeau E., Brunon C., Degraeve P., Léonard D., Grossiord C., Bessueille F., Cottaz A., Renaud F., Ferreira I., Darroux C. (2012). Evaluation of antimicrobial activity of a polyhexamethylene biguanide-coated textile by monitoring both bacterial growth (iso 20743/2005 standard) and viability (live/dead baclight kit). J. Food Saf..

[36] Sciessent Agion. http://www.sciessent.com/agion-antimicrobial-technology.

[37] Chemical M. Antimicrobial-Alphasan-Additive. http://millikenchemical.com/milliken-antimicrobial-alphasan-additive/2016.

[38] Gettings R.L., Triplett B.L. (1978). A new durable antimicrobial finish for textiles. Book of Papers.

[39] Prospector^®^. Cosmocil CQ™. https://www.ulprospector.com/en/eu/PersonalCare/Detail/762/30958/Cosmocil-CQ.

[40] Microban^®^. http://www.microban.com/en-uk.

[41] Lonza Reputex™ Antimicrobials. http://www.lonza.com/products-services/industrial-solutions/materials-protection/reputex-antimicrobials.aspx.

[42] Industries LNC Cotton-Products. http://www.lnchemicals.com/cotton-products2.html.

[43] SANITIZED Technology for Textiles. http://www.sanitized.com/technology-for-textiles/2016.

[44] Associates TR Ultra-Fresh. http://www.ultra-fresh.com.

[45] Company, TDC. Silvadur. http://www.dow.com/silvadur.

[46] PURE Bioscience I. Silvérion 2400. http://www.purebio.com/products/silverion-2400.htm.

[47] Jones R.D., Jampani H.B., Newman J.L., Lee A.S. (2000). Triclosan: A review of effectiveness and safety in health care settings. Am. J. Infect. Control.

[48] Mansfield R.G. (2002). Keeping it fresh. Text. World.

[49] Russell A. (2004). Bacterial adaptation and resistance to antiseptics, disinfectants and preservatives is not a new phenomenon. J. Hosp. Infect..

[50] Latch D.E., Packer J.L., Arnold W.A., McNeill K. (2003). Photochemical conversion of triclosan to 2, 8-dichlorodibenzo-p-dioxin in aqueous solution. J. Photochem. Photobiol. A Chem..

[51] Buth J.M., Steen P.O., Sueper C., Blumentritt D., Vikesland P.J., Arnold W.A., McNeill K. (2010). Dioxin photoproducts of triclosan and its chlorinated derivatives in sediment cores. Environ. Sci. Technol..

[52] Hasan J., Crawford R.J., Ivanova E.P. (2013). Antibacterial surfaces: The quest for a new generation of biomaterials. Trends Biotechnol..

[53] Dastjerdi R., Montazer M. (2010). A review on the application of inorganic nano-structured materials in the modification of textiles: Focus on anti-microbial properties. Colloids Surf. B Biointerfaces.

[54] Raghupathi K.R., Koodali R.T., Manna A.C. (2011). Size-dependent bacterial growth inhibition and mechanism of antibacterial activity of zinc oxide nanoparticles. Langmuir.

[55] Farouk A., Moussa S., Ulbricht M., Textor T. (2012). Zno nanoparticles-chitosan composite as antibacterial finish for textiles. Int. J. Carbohydr. Chem..

[56] NanoHorizons I. Smartsilver. http://www.smartsilver.com.

[57] Morais D., Rodrigues M., Lopes M., Coelho M., Maurício A.C., Gomes R., Amorim I., Ferraz M., Santos J., Botelho C. (2013). Biological evaluation of alginate-based hydrogels, with antimicrobial features by ce (iii) incorporation, as vehicles for a bone substitute. J. Mater. Sci. Mater. Med..

[58] Badawy M.E., Rabea E.I. (2011). A biopolymer chitosan and its derivatives as promising antimicrobial agents against plant pathogens and their applications in crop protection. Int. J. Carbohydr. Chem..

[59] Wang X., Du Y., Fan L., Liu H., Hu Y. (2005). Chitosan-metal complexes as antimicrobial agent: Synthesis, characterization and structure-activity study. Polym. Bull..

[60] Knittel D., Schollmeyer E. (2006). Chitosans for permanent antimicrobial finish on textiles. Lenzing. Ber..

[61] Lim S.-H., Hudson S.M. (2004). Application of a fiber-reactive chitosan derivative to cotton fabric as an antimicrobial textile finish. Carbohydr. Polym..

[62] Zhou C., Wang M., Zou K., Chen J., Zhu Y., Du J. (2013). Antibacterial polypeptide-grafted chitosan-based nanocapsules as an “armed” carrier of anticancer and antiepileptic drugs. ACS Macro Lett..

[63] Kong M., Chen X.-G., Xue Y.-P., Liu C.-S., Yu L.-J., Ji Q.-X., Cha D.S., Park H.J. (2008). Preparation and antibacterial activity of chitosan microshperes in a solid dispersing system. Front. Mater. Sci. China.

[64] No H.K., Kim S.H., Lee S.H., Park N.Y., Prinyawiwatkul W. (2006). Stability and antibacterial activity of chitosan solutions affected by storage temperature and time. Carbohydr. Polym..

[65] Shahid M., Mohammad F. (2013). Perspectives for natural product based agents derived from industrial plants in textile applications—A review. J. Clean. Prod..

[66] Cowan M.M. (1999). Plant products as antimicrobial agents. Clin. Microbiol. Rev..

[67] Venugopala K.N., Rashmi V., Odhav B. (2013). Review on natural coumarin lead compounds for their pharmacological activity. BioMed Res. Int..

[68] Kheeree N., Sangvanich P., Puthong S., Karnchanatat A. (2010). Antifungal and antiproliferative activities of lectin from the rhizomes of curcuma amarissima roscoe. Appl. Biochem. Biotechnol..

[69] Sobczak M., Dębek C., Olędzka E., Kozłowski R. (2013). Polymeric systems of antimicrobial peptides—Strategies and potential applications. Molecules.

[70] Gouveia I.C. (2010). Nanobiotechnology: A new strategy to develop non-toxic antimicrobial textiles. Current Research, Technology and Education Topics in Applied Microbiology and Microbial Biotechnology.

[71] Rokitskaya T.I., Kolodkin N.I., Kotova E.A., Antonenko Y.N. (2011). Indolicidin action on membrane permeability: Carrier mechanism *versus* pore formation. Biochim. Biophys. Acta BBA Biomembr..

[72] Breidenstein E.B., Courvalin P., Meziane-Cherif D. (2015). Antimicrobial activity of plectasin nz2114 in combination with cell wall targeting antibiotics against vana-type enterococcus faecalis. Microb. Drug Resist..

[73] Kyung K., Lee Y. (2001). Antimicrobial activities of sulfur compounds derived from s-alk (en) yl-l-cysteine sulfoxides in allium and brassica. Food Rev. Int..

[74] Bach S.M., Fortuna M.A., Attarian R., de Trimarco J.T., Catalán C., Av-Gay Y., Bach H. (2011). Antibacterial and cytotoxic activities of the sesquiterpene lactones cnicin and onopordopicrin. Nat. Prod. Commun..

[75] Mathabe M.C., Hussein A.A., Nikolova R.V., Basson A.E., Meyer J.M., Lall N. (2008). Antibacterial activities and cytotoxicity of terpenoids isolated from spirostachys africana. J. Ethnopharmacol..

[76] Petnual P., Sangvanich P., Karnchanatat A. (2010). A lectin from the rhizomes of turmeric (*Curcuma longa* L.) and its antifungal, antibacterial, and α-glucosidase inhibitory activities. Food Sci. Biotechnol..

[77] Orhan D.D., Özçelik B., Özgen S., Ergun F. (2010). Antibacterial, antifungal, and antiviral activities of some flavonoids. Microbiol. Res..

[78] Rattanachaikunsopon P., Phumkhachorn P. (2010). Contents and antibacterial activity of flavonoids extracted from leaves of psidium guajava. J. Med. Plants Res..

[79] Özçelik B., Orhan D.D., Özgen S., Ergun F. (2008). Antimicrobial activity of flavonoids against extended-spectrum β-lactamase (esβl)-producing *Klebsiella pneumoniae*. Trop. J. Pharm. Res..

[80] Ignacimuthu S., Pavunraj M., Duraipandiyan V., Raja N., Muthu C. (2009). Antibacterial activity of a novel quinone from the leaves of pergularia daemia (forsk.), a traditional medicinal plant. Asian J. Tradit. Med..

[81] Singh D., Verma N., Raghuwanshi S., Shukla P., Kulshreshtha D. (2006). Antifungal anthraquinones from saprosma fragrans. Bioorg. Med. Chem. Lett..

[82] Engels C., Knodler M., Zhao Y.-Y., Carle R., Gänzle M.G., Schieber A. (2009). Antimicrobial activity of gallotannins isolated from mango (mangifera indica l.) kernels. J. Agric. Food Chem..

[83] Scalbert A. (1991). Antimicrobial properties of tannins. Phytochemistry.

[84] Saleem M., Nazir M., Ali M.S., Hussain H., Lee Y.S., Riaz N., Jabbar A. (2010). Antimicrobial natural products: An update on future antibiotic drug candidates. Nat. Prod. Rep..

[85] Hui F., Debiemme-Chouvy C. (2013). Antimicrobial N-halamine polymers and coatings: A review of their synthesis, characterization, and applications. Biomacromolecules.

[86] Ren X., Kou L., Kocer H.B., Zhu C., Worley S., Broughton R., Huang T. (2008). Antimicrobial coating of an N-halamine biocidal monomer on cotton fibers via admicellar polymerization. Colloids Surf. A Physicochem. Eng. Asp..

[87] Cao Z., Sun Y. (2008). N-halamine-based chitosan: Preparation, characterization, and antimicrobial function. J. Biomed. Mater. Res. A.

[88] Aflori M., Drobotă M., Ţîmpu D., Bărboiu V. Amine functionality of poly(ethylene terepthalate) films surfaces induced by chemical and rf plasma treatments. Proceedings of the 28th ICPIG (International Conference on Phenomena in Ionized Gases).

[89] Wei Q. (2009). Surface Modification of Textiles.

[90] Davis R., El-Shafei A., Hauser P. (2011). Use of atmospheric pressure plasma to confer durable water repellent functionality and antimicrobial functionality on cotton/polyester blend. Surf. Coat. Technol..

[91] Lam Y., Kan C., Yuen C. (2013). A study of metal oxide on antimicrobial effect of plasma pre-treated cotton fabric. Fibers Polym..

[92] Caillier L., de Givenchy E.T., Levy R., Vandenberghe Y., Géribaldi S., Guittard F. (2009). Synthesis and antimicrobial properties of polymerizable quaternary ammoniums. Eur. J. Med. Chem..

[93] Lu G., Wu D., Fu R. (2007). Studies on the synthesis and antibacterial activities of polymeric quaternary ammonium salts from dimethylaminoethyl methacrylate. React. Funct. Polym..

[94] Nalwa H.S. (2003). Handbook of Organic-Inorganic Hybrid Materials and Nanocomposites.

[95] Son Y.A., Sun G. (2003). Durable antimicrobial nylon 66 fabrics: Ionic interactions with quaternary ammonium salts. J. Appl. Polym. Sci..

[96] Kawabata A., Taylor J.A. (2004). Effect of reactive dyes upon the uptake and antibacterial action of poly (hexamethylene biguanide) on cotton. Part 1: Effect of bis (monochlorotriazinyl) dyes. Color. Technol..

[97] Kawabata A., Taylor J. (2006). The effect of reactive dyes upon the uptake and anti bacterial action of poly (hexamethylene biguanide) on cotton. Part 2: Uptake of poly (hexamethylene biguanide) on cotton dyed with β-sulphatoethylsulphonyl reactive dyes. Dyes Pigments.

[98] Blackburn R.S., Harvey A., Kettle L.L., Payne J.D., Russell S.J. (2006). Sorption of poly (hexamethylenebiguanide) on cellulose: Mechanism of binding and molecular recognition. Langmuir.

[99] Badrossamay M.R., Sun G. (2008). Acyclic halamine polypropylene polymer: Effect of monomer structure on grafting efficiency, stability and biocidal activities. React. Funct. Polym..

[100] Ulman K.N., Shukla S.R. (2015). Admicellar polymerization and its application in textiles. Adv. Polym. Technol..

[101] Cerkez I., Kocer H.B., Worley S., Broughton R., Huang T. (2011). N-halamine biocidal coatings via a layer-by-layer assembly technique. Langmuir.

[102] Carré A., Mittal K.L. (2011). Surface and Interfacial Aspects of Cell Adhesion.

[103] Sedlarik V. (2013). Antimicrobial modifications of polymers. Biodegradation-Life of Science.

[104] Bhardwaj N., Kundu S.C. (2010). Electrospinning: A fascinating fiber fabrication technique. Biotechnol. Adv..

[105] Mirjalili M., Zohoori S. (2016). Review for application of electrospinning and electrospun nanofibers technology in textile industry. J. Nanostruct. Chem..

[106] Lagaron J.M., Ocio M.J., Lopez-Rubio A. (2011). Antimicrobial Polymers.

[107] Nephew S. Pro-Acticoat. http://feridas.smith-nephew.pt/pro-acticoat.html.

[108] Sinterama Bactershield^®^. http://www.sinterama.com/site/app01/lng/eng/public.nsf/content?openagent&grp=3&act=prd&sec=3&psp=WCNBREFS_000009&imp=.

[109] Trevira Trevira Bioactive. http://www.trevira.com/en/textiles-made-from-trevira/brands/trevira-bioactive.html.

[110] Crabyon. http://www.swicofil.com/crabyon.html.

[111] Toyobo Products. http://www.toyobo-global.com/products/#?tab=3.

[112] Medtronic Kendall™ AMD Antimicrobial foam Dressings. http://www.kendallamdfoam.com/pages.aspx?page=HowItWorks.

[113] Seacell. http://www.smartfiber.info/seacell.

[114] Slater K. (2003). Environmental Impact of Textiles: Production, Processes and Protection.

[115] United States Environmental Protection Agency (2008). Reregistration Eligibility Decision for Triclosan. https://archive.epa.gov/pesticides/reregistration/web/pdf/2340red.pdf.

[116] Eckelman M.J., Graedel T. (2007). Silver emissions and their environmental impacts: A multilevel assessment. Environ. Sci. Technol..

[117] Kan C.-W. (2014). A Novel Green Treatment for Textiles: Plasma Treatment as a Sustainable Technology.

[118] Kasiri M.B., Safapour S. (2014). Natural dyes and antimicrobials for green treatment of textiles. Environ. Chem. Lett..

[119] Kalia S., Thakur K., Celli A., Kiechel M.A., Schauer C.L. (2013). Surface modification of plant fibers using environment friendly methods for their application in polymer composites, textile industry and antimicrobial activities: A review. J. Environ. Chem. Eng..

[120] Shahid M., Mohammad F. (2013). Green chemistry approaches to develop antimicrobial textiles based on sustainable biopolymers—A review. Ind. Eng. Chem. Res..

[121] Hu S., Hsieh Y.-L. (2015). Synthesis of surface bound silver nanoparticles on cellulose fibers using lignin as multi-functional agent. Carbohydr. Polym..

[122] Khurshid M.F., Ayyoob M., Asad M., Shah S.N.H. (2015). Assessment of eco-friendly natural antimicrobial textile finish extracted from aloe vera and neem plants. FIBRES TEXTILES East. Eur..

[123] Radhouani H., Silva N., Poeta P., Torres C., Correia S., Igrejas G. (2014). Potential impact of antimicrobial resistance in wildlife, environment and human health. Front. Microbiol..

[124] Reidy B., Haase A., Luch A., Dawson K.A., Lynch I. (2013). Mechanisms of silver nanoparticle release, transformation and toxicity: A critical review of current knowledge and recommendations for future studies and applications. Materials.

[125] Randall C.P., Gupta A., Jackson N., Busse D., O’Neill A.J. (2015). Silver resistance in gram-negative bacteria: A dissection of endogenous and exogenous mechanisms. J. Antimicrob. Chemother..

[126] Heuer O.E., Kruse H., Grave K., Collignon P., Karunasagar I., Angulo F.J. (2009). Human health consequences of use of antimicrobial agents in aquaculture. Clin. Infect. Dis..

[127] World Health Organization (2014). Antimicrobial Resistance: Global Report on Surveillance.

[128] Lansdown A. (2006). Silver in health care: Antimicrobial effects and safety in use. Curr. Probl. Dermatol..

[129] Wollina U., Abdel-Naser M., Verma S. (2006). Skin physiology and textiles–consideration of basic interactions. Curr. Probl. Dermatol..

